# Comprehensive profiling of novel microRNA-9 targets and a tumor suppressor role of microRNA-9 via targeting IGF2BP1 in hepatocellular carcinoma

**DOI:** 10.18632/oncotarget.5969

**Published:** 2015-10-19

**Authors:** Jiangbo Zhang, Jin Cheng, Zhenzhen Zeng, Yongfeng Wang, Xiaojun Li, Qing Xie, Junqiao Jia, Ying Yan, Zhengyang Guo, Jian Gao, Mingjie Yao, Xiangmei Chen, Fengmin Lu

**Affiliations:** ^1^ Department of Microbiology & Infectious Disease Center, School of Basic Medical Sciences, Peking University Health Science Center, Beijing 100191, China; ^2^ Sun Yat-sen University Cancer Center, State Key Laboratory of Oncology in South China, Collaborative Innovation Center for Cancer Medicine, Guangzhou 510060, China; ^3^ Institute of Sports Medicine, Beijing Key Laboratory of Sports Injuries, Peking University Third Hospital, Beijing 100191, China; ^4^ Department of Clinical Laboratory, Beijing Shijitan Hospital, Capital Medical University, Beijing 100038, China

**Keywords:** microRNA-9, hepatocellular carcinoma, hypermethylation, IGF2BP1, AKT&ERK

## Abstract

MicroRNA-9 (miR-9) dysregulation is implicated in a variety of human malignancies including hepatocellular carcinoma (HCC), but its role remains contradictory. In this study, we explored the expression and methylation status of miR-9 in HCC samples, as well as the tumor-related functions of miR-9 *in vitro*. Bioinformatics analysis, array-based RNA expression profile, and literature retrieval were used to identify miR-9 targets in HCC. The potential downstream candidates were then validated by luciferase reporter assay, real-time quantitative PCR, and western blot or enzyme linked immunosorbent assay (ELISA). The expression status and clinicopathologic significances of miR-9 target genes in clinical samples were further explored. The results showed that miR-9 was frequently downregulated in primary HCC. Its silencing was largely contributed by a high frequency (42.5%) of mir-9-1 hypermethylation, which was correlated with bigger tumor size (*P* = 0.0234). *In vitro* functional studies revealed that miR-9 restoration retarded HCC cell proliferation and migration. IL-6, AP3B1, TC10, ONECUT2, IGF2BP1, MYO1D, and ANXA2 were confirmed to be miR-9 targets in HCC. Among them, ONECUT2, IGF2BP1, and ANXA2 were confirmed to be aberrantly upregulated in HCC. Moreover, upregulation of ONECUT2, IGF2BP1, and IL-6 were significantly associated with poor post-surgery prognosis (*P* = 0.0458, *P* = 0.0037 and *P* = 0.0461, respectively). Mechanically, miR-9 plays a tumor suppressive role partially through a functional miR-9/IGF2BP1/AKT&ERK axis. Our study suggests that miR-9 functions as a tumor suppressor in HCC progression by inhibiting a series of target genes, including the newly validated miR-9/IGF2BP1/AKT&ERK axis, thus providing potential therapeutic targets and novel prognostic biomarkers for HCC patients.

## INTRODUCTION

Hepatocellular carcinoma (HCC) is one of the most prevalent malignancies and ranks the third leading cause of cancer-related deaths worldwide [1]. The morbidity rate of HCC has gradually increased in recent decades and the prognosis remains poor due to tumor progression and high tumor recurrence rate [2]. There are no well-established effective adjuvant therapies for HCC currently, therefore, new researches should be focused on developing more therapeutic strategies against HCC so as to effectively improve patients' survival outcome. The difficulties of treating HCC are mainly due to an incomplete understanding of the heterogeneous genetic and epigenetic alterations of HCC. Therefore discovering new molecular carcinogenic mechanisms of HCC will give guidance for the development of novel clinical treatment.

MicroRNAs are endogenous, small non-coding RNA molecules with a length of 18–25 nucleotides. Its deregulation has been widely reported to be involved in the development of various cancers [3, 4]. Recent studies have revealed that microRNA-9 (miR-9) is aberrantly expressed in many cancer types including breast cancer, colorectal cancer, lung cancer, etc. In the human genome there are three miR-9 genes (mir-9-1 on chromosome 1, mir-9-2 on chromosome 5 and mir-9-3 on chromosome 15) with an identical mature miR-9 sequence [5]. The methylation of hsa-mir-9-1 was first described in human breast cancer [6], and it was later demonstrated to be one of the most frequently methylated microRNAs in various human malignancies [7, 8]. The role of miR-9 in cancers remains controversial. It has been shown to be either an oncogenic microRNA or a tumor suppressor depending on different tissue types and downstream targets. For instance, miR-9 has been shown to target NF-κB in ovarian cancer and gastric cancer leading to inhibition of cell proliferation and metastasis [9, 10]. On the other hand, some groups have also discovered its oncogenic function: miR-9 overexpression has been shown to enhance metastasis in esophageal squamous cell carcinoma by targeting E-cadherin [11]. In cervical cancer, human papillomavirus (HPV)-induced miR-9 activation led to significantly increased cell motility by downregulating FSTL1 and ALCAM [12].

In this study, we investigated the aberrant status of miR-9 and its potential target genes to explore this microRNA's cancer-related functions in HCC. Our results revealed the efficient tumor suppressive role of miR-9 in HCC, and more importantly, the newly identified miR-9 targets might serve as potential therapeutic targets and novel prognosis indicators in HCC.

## RESULTS

### Methylation of miR-9 contributed to its aberrant down-regulation in HCC

**Figure 1:**
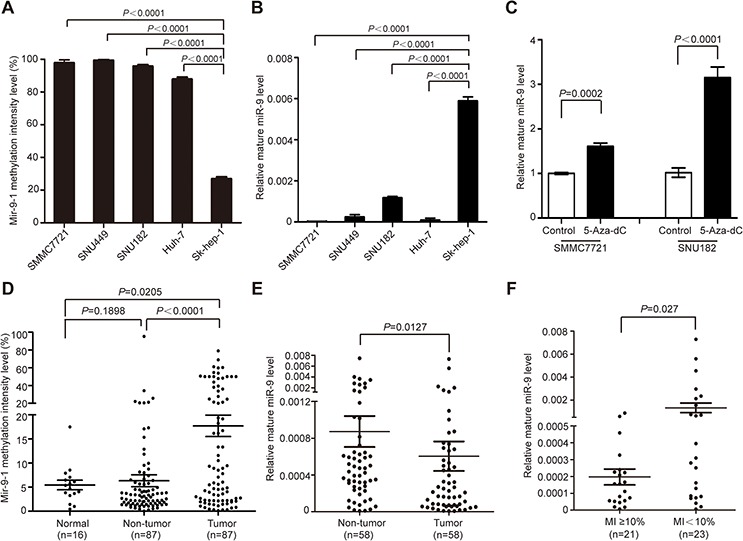
Methylation contributed to miR-9 aberrant down-regulation in HCC **A.** The methylation intensity (MI) of mir-9-1 in SMMC7721, SNU449, SNU182, Huh-7 and Sk-hep-1. **B.** The relative expression level of miR-9 in 5 HCC cell lines (mean ± SEM; *N* = 3). **C.** The relative miR-9 level in SMMC7721 and SNU182 before and after demethylation treatment using 5-aza-2′-deoxycytidine (mean ± SEM; *N* = 3). **D.** The methylation intensity (MI) of mir-9-1 in 87 pairs of HCC tumor tissues and adjacent non-tumor tissues and 16 healthy live tissues. Data were presented as mean ± SEM. **E.** The relative expression level of miR-9 in 58 pairs of HCC tumor tissues and adjacent non-tumor tissues. Data were presented as mean ± SEM. **F.** The expression level of miR-9 in methylated group (MI ≥ 10%) and unmethylated group (MI < 10%). Data were presented as mean ± SEM. Significant differences were determined using Student *t* tests.

In previous studies, miR-9 was reported to be methylated in various tumors including HCC. To investigate whether methylation caused miR-9 downregulation in HCC, firstly, the methylation intensity (MI) of all three members of miR-9 family (mir-9-1, 2 and 3) was detected in SMMC7721, SNU449, SNU182, Huh-7, and Sk-hep-1 (adenocarcinoma originated) cells. The results showed that mir-9-1 was extensively methylated in all cell lines, with the exception for Sk-hep-1, which was moderately methylated (Figure 1A). As for mir-9-2 and mir-9-3, no methylation was detected (data not shown). Concordantly, the expression level of miR-9 was significantly higher in Sk-hep-1 than in the other four cell lines (Figure 1B). Furthermore, demethylation treatment of cultured SMMC7721 and SNU182 cells using 5-aza-2′-deoxycytidine restored miR-9 expression in both cells (Figure 1C). The above results suggested that hypermethylation of mir-9-1 might be a main contributor to its down-regulation in HCC.

To confirm this postulation, we then tested the methylation status of mir-9-1 in 87 pairs of primary HCC tissues and 16 healthy liver donors, and found that the frequency of mir-9-1 gene hypermethylation (MI ≥ 10%) in tumor tissues (37/87) was significantly higher than that in paired adjacent non-tumor tissues (12/87) (42.5% *vs*. 13.8%, *P* = 0.0097). Concordantly, statistical analysis revealed that the methylation intensity of mir-9-1 in tumor tissues was significantly higher than that of in non-tumor tissues (*P* < 0.0001) and normal liver tissues (*P* = 0.0205) (Figure 1D). Next, we detected the expression level of miR-9 in 58 pairs of human primary HCC tissues and the adjacent non-tumor liver tissues by real-time qPCR. The result showed that the expression of miR-9 in tumor tissues was significantly lower than that of in non-tumor tissues (*P* = 0.0127, Figure 1E). To further demonstrate the negative correlation between mir-9-1 methylation and miR-9 expression, we divided the 58 tumor samples into methylated group (MI ≥ 10%) and unmethylated group (MI < 10%) according to mir-9-1 methylation status as we previously described [13]. As expected, the expression level of miR-9 was significantly lower in the methylated group than that of in the unmethylated group (*P* = 0.027) (Figure 1F). Moreover, the methylation status of mir-9-1 was positively correlated with larger tumor size (*P* = 0.0234) (Table 1), suggesting the increase of mir-9-1 methylation intensity might contribute to HCC tumor growth.

**Table 1:** The correlation between mir-9-1 promoter methylation status in HCC tissues and patients clinicopathological features

Clinicopathological features		Hypermethylated	Unmethylated	*P*
		(*n* = 37)	(*n* = 48)	
**Gender**	Male	31	37	*0.444*
	Female	6	11	
**Age**	≥50	21	28	*0.884*
	<50	16	20	
**Cirrhosis**	Yes	33	43	*0.763*
	No	4	3	
	N/A	0	2	
**Portal Vein Invasion**	Present	11	15	*0.779*
	Absent	26	31	
	N/A	0	2	
**Tumor size**	≥5 cm	29	31	***0.023***
	<5 cm	3	14	
	N/A	5	3	
**Tumor Encapsulation**	Complete	31	37	*0.948*
	Incomplete	4	5	
	N/A	2	6	
**Intrahepatic Dissemination**	Present	18	16	*0.202*
	Absent	19	30	
	N/A	0	2	
**AFP**	<400	16	24	*0.363*
	≥400	21	21	
	N/A	0	3	
**Virus infection background**	HBV	31	41	*1.000*
	HCV	0	2	
	Others	6	5	
**HBsAg**	Positive	31	41	*0.750*
	Negative	6	6	
	N/A	0	1	
**HBeAg**	Positive	1	1	*1.000*
	Negative	35	46	
	N/A	1	1	
**BCLC**	A	11	21	0.173
	B+C	25	24	
	N/A	1	3	

Note: The number of patients was not identical in all features due to clinical data availability.

*P*-values were calculated by chi-square test or Fisher's exact test. *P*-value of < 0.05 (two sided) was considered as significant and written in bold text.

### Ectopic miR-9 expression inhibited HCC oncogenic properties *in vitro*

**Figure 2:**
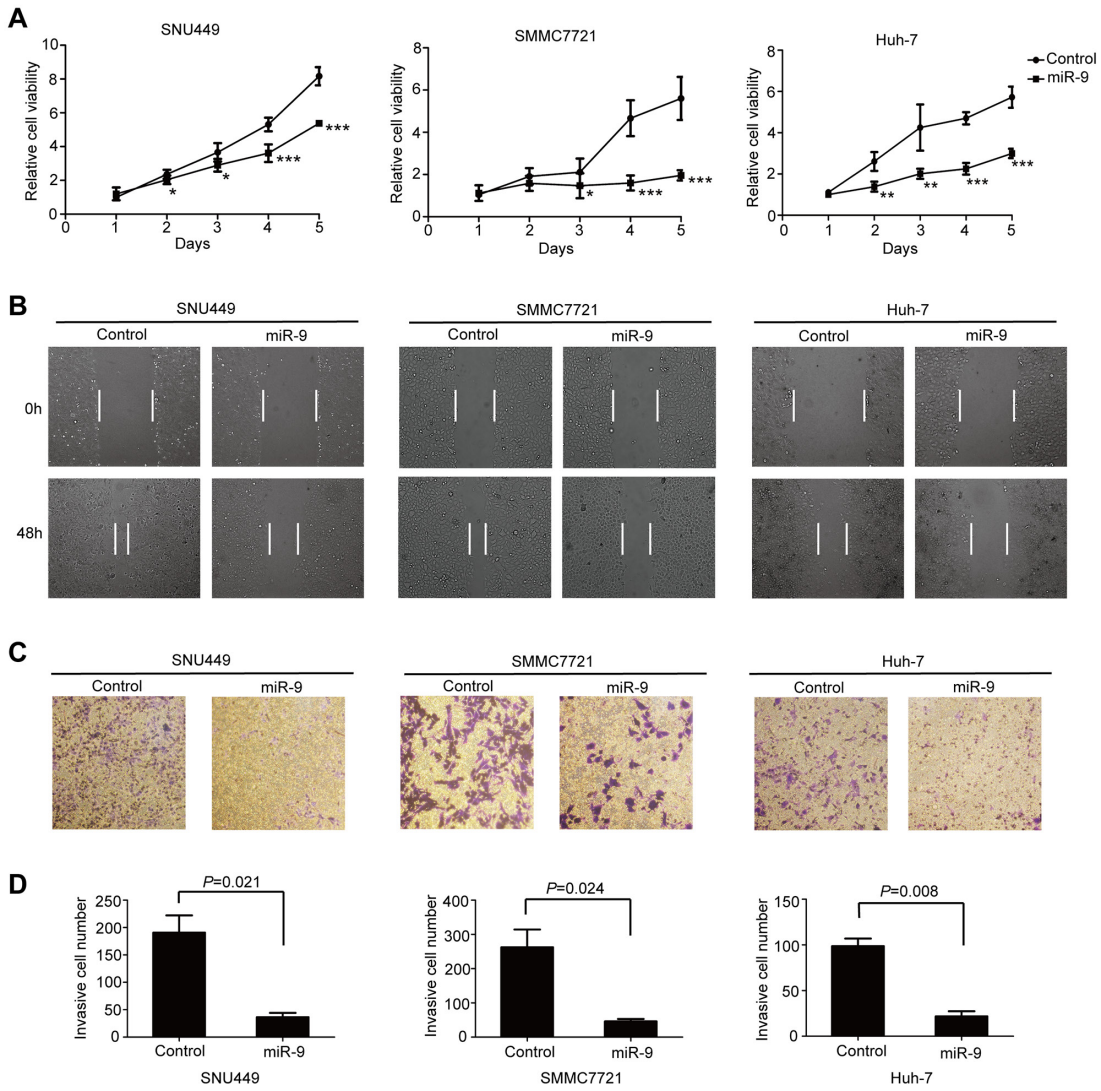
MiR-9 inhibited HCC oncogenic properties *in vitro* **A.** The growth curve of miR-9 overexpressing HCC cells and control cells. Data were presented as mean ± SEM. *P* values < 0.05 (*), < 0.01 (**) or < 0.001 (***) were indicated (Student's *t*-test). **B.** Representative images of wound healing assay showing the effects of miR-9 on HCC cell migration. **C.** Representative images of transwell assay showing the effect of miR-9 on HCC cell migration. **D.** The statistical analysis of (C).

To further confirm our inference that miR-9 might suppress HCC tumor growth, SNU449, SMMC7721 and Huh-7 cell lines were used to determine the effects of miR-9 restoration on HCC oncogenic properties. As expected, re-expression of miR-9 significantly inhibited cell proliferation in all three cell lines (Figure 2A). Wound healing assay and transwell migration assay were conducted to explore the influence of miR-9 on HCC cell motility. The results showed that migratory cells were dramatically decreased in ectopic miR-9 expressing cell lines (Figure 2B–2D). These observations indicated that miR-9 not only inhibited HCC cell proliferation, but also significantly suppressed the migratory ability of HCC cells.

### Preliminary screening for novel miR-9 targets in HCC

**Figure 3:**
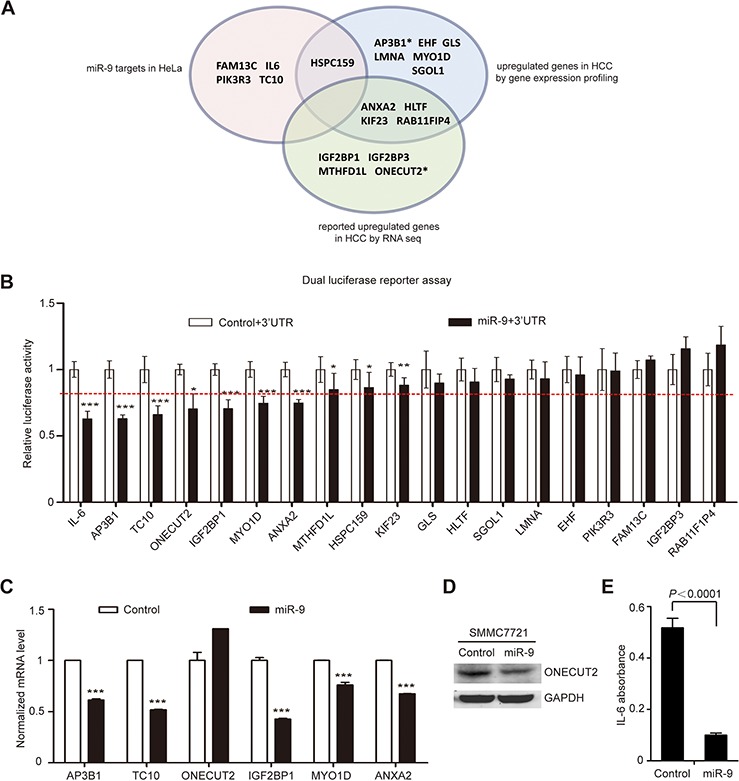
Comprehensive screening for miR-9 downstream target in HCC **A.** Systematic identification of miR-9 targets based on multiple selection strategies. The pink area indicates miR-9 target genes confirmed in HeLa cells; the blue area indicates upregulated genes in 6 pairs of primary HCC tissues by gene expression profiling; the green area indicates previously reported upregulated genes in HCC by RNA sequencing. *represents the previously reported target genes of miR-9. **B.** The results of luciferase activity detection. ‘miR-9+3′UTR’ and ‘control+3′UTR’ means SMMC7721 cells co-transfected with pGL3-Luc-3′-UTR-WT and U6-mir-9-1 or U6-control vector (see Materials and Methods section for details). The data represents the mean ± SEM of 3 independent experiments done in 9 repeats. The red dotted line indicates the 20% suppression rate. **C.** Real-time RT-PCR results of validating the relative mRNA level change of potential miR-9 target genes in SMMC7721 (mean ± SEM; *N* = 3). **D.** Western blot results of detecting ONECUT2 protein levels before and after miR-9 expression in SMMC7721. GAPDH was used as a loading control. **E.** ELISA results of measuring the concentration of IL-6 in the SMMC7721 cell supernatant before and after ectopic expression of miR-9 (mean ± SEM; *N* = 3). *P* values < 0.05 (*), < 0.01 (**) or < 0.001 (***).

For the purpose of more comprehensive screening for novel miR-9 targets, we overlapped the miR-9 target gene list predicted by bioinformatics analysis (2414 genes, Supplementary Table S1) with gene lists acquired using three different strategies: (1) upregulated genes in 6 pairs of primary HCC tissues by gene expression profiling (Supplementary Table S2); (2) previously reported upregulated genes in HCC [14]; (3) miR-9 target genes confirmed in HeLa cells by our lab [15]. Meanwhile, to further narrow down the range of potential candidates, the functions of candidate genes have also been taken into consideration. Finally, 19 potential target genes (IL-6, AP3B1, TC10, ONECUT2, IGF2BP1, MYO1D, ANXA2, MTHFD1L, HSPC159, KIF23, GLS, HLTF, SGOL1, LMNA, EHF, PIK3R3, FAM13C, IGF2BP3, and RAB11F1P4) were chosen for further validation (Figure 3A).

To confirm the above predictions, the 3′-UTR of the 19 candidate genes containing putative miR-9 binding site were amplified by PCR and inserted into the downstream multiple cloning site of *firefly* luciferase gene in the pGL3 vector, and then luciferase reporter assay was performed by co-transfecting the above constructs with U6-mir-9-1 precursor or U6 control plasmid into SMMC7721 cells. The result showed that 7 of them (IL-6, AP3B1, TC10, ONECUT2, IGF2BP1, MYO1D and ANXA2) presented reduced luciferase activities (up to 20% suppression rate) when miR-9 was overexpressed (Figure 3B). Among these genes, AP3B1, ONECUT2 and IL-6 were already confirmed to be direct targets of miR-9 in breast cancer, insulin-producing cells, and cervical adenocarcinoma, respectively [15–17], which demonstrated the reliability of our screening strategy. Next, real-time RT-PCR revealed that miR-9 significantly suppressed the endogenous mRNA expression of 5 genes (AP3B1, TC10, IGF2BP1, MYO1D and ANXA2) in SMMC7721, but did not inhibit the mRNA level of ONECUT2 (Figure 3C). Since microRNAs function in not only mRNA degradation but also translational repression, we proceeded to detect ONECUT2 protein level change after miR-9 overexpression. Indeed, ONECUT2 protein level was found decreased in SMMC7721 cells overexpressing miR-9 (Figure 3D). We also performed ELISA to directly detect the secretion of IL-6 in the cell supernatant after miR-9 overexpression. It showed that miR-9 greatly suppressed IL-6 expression in SMMC7721 (Figure 3E). The above results indicated that IL-6, AP3B1, TC10, ONECUT2, IGF2BP1, MYO1D and ANXA2 are potential target genes of miR-9 in HCC.

### The expression status of validated miR-9 targets in HCC tissues

**Figure 4:**
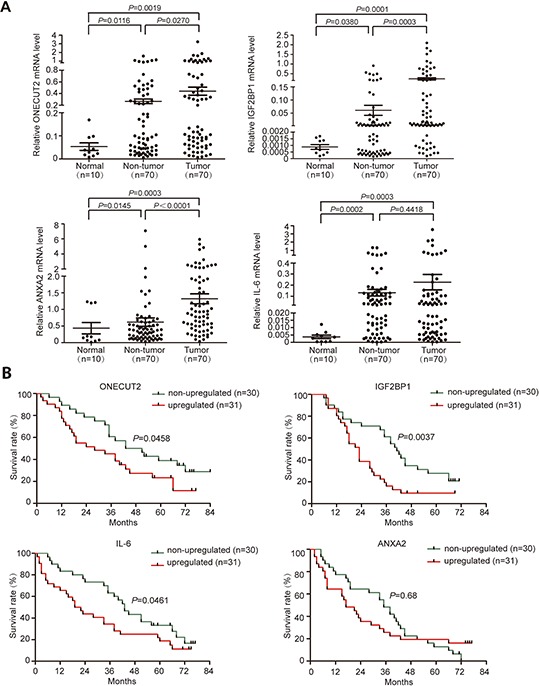
Validation of miR-9 targets expression level in HCC tissues **A.** Relative mRNA levels of ONECUT2, IGF2BP1, ANXA2, and IL-6 genes in 70 paired HCC tumor tissues and adjacent non-tumor tissues, as well as 10 normal tissues. Data were presented as mean ± SEM. **B.** Prognostic significance of ONECUT2, IGF2BP1, IL-6 and ANXA2 expression status in HCC.

In order to further explore the expression status of these 7 candidate genes in clinical samples, their mRNA expression levels were detected in 30 pairs of HCC tissues and adjacent non-tumor tissues, as well as 10 normal tissues. Among all genes tested, ONECUT2, IGF2BP1, and ANXA2 were aberrantly upregulated in tumor tissues compared to non-tumor tissues (Supplementary Figure S1A). IL-6 was upregulated in both tumor and adjacent non-tumor tissues compared to normal tissues (Supplementary Figure S1A). However, TC10 was significantly downregulated in both tumor and adjacent non-tumor tissues compared to normal tissues, and no difference was found between the former two groups (Supplementary Figure S1B). As for AP3B1 and MYO1D, no significant difference was found between any of the three groups (Supplementary Figure S1B). Next, we proceeded to enlarge the sample number to 70 to validate the expression status of ONECUT2, IGF2BP1, ANXA2, and IL-6 in HCC. The result confirmed that the expression levels of ONECUT2, IGF2BP1, and ANXA2 were significantly elevated in non-tumor tissues compared to normal tissues (*P* = 0.0116, *P* = 0.0380 and *P* = 0.0145, respectively), and further elevated in HCC tissues (*P* = 0.0270, *P* = 0.0003 and *P* < 0.0001, respectively) (Figure 4A). On the other hand, the mRNA levels of IL-6 in tumor and adjacent non-tumor tissues were both significantly higher than normal controls (*P* = 0.0003 and *P* = 0.0002, respectively) (Figure 4A). The relatively high level of IL-6 in non-tumor tissues might be caused by the infiltration of inflammatory cells.

We next analyzed the correlation between the expression status of ONECUT2, IGF2BP1, ANXA2 and IL-6 with HCC patient's clinicopathological features. The results showed that an increase of ONECUT2 expression happened more frequently in male patients (*P* = 0.013) and patients infected with HBV (*P* = 0.024) (Supplementary Table S3). Intriguingly, higher IGF2BP1 mRNA level in tumor was positively correlated with higher level of α-fetoprotein (AFP), the most widely used diagnostic biomarker for HCC [18] (*P* = 0.029) (Supplementary Table S3). Moreover, upregulation of IL-6 in HCC tissues was positively correlated with a later BCLC stage (*P* = 0.013) (Supplementary Table S3). As for ANXA2, no clinicopathological significance was found.

To understand the prognostic significance of aberrantly up-regulated ONECUT2, IGF2BP1, ANXA2, and IL-6 in HCC, we analyzed the correlation between these genes' expression status and patients' overall post-surgery survival period. The results showed that upregulation of ONECUT2, IGF2BP1, and IL-6 were all significantly correlated with poor post-surgery survival (*P* = 0.0458, *P* = 0.0037, and *P* = 0.0461, respectively; Figure 4B). As for ANXA2, no prognostic significance was found in our current study (Figure 4B). These results indicated that the expression status of ONECUT2, IGF2BP1, and IL-6 in HCC might be able to be used as potential prognosis indexes to predict patient survival.

### MiR-9 inhibited ERK and AKT pathway through targeting IGF2BP1 in HCC

**Figure 5:**
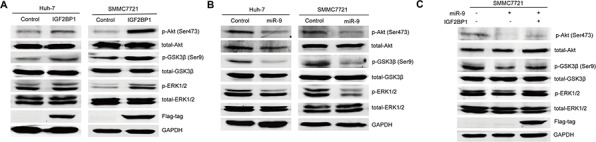
The suppressive function of miR-9 for ERK and AKT pathways by targeting IGF2BP1 in HCC **A.** Western blot result of indicated protein level before and after IGF2BP1 expression in Huh-7 and SMMC7721. GAPDH was used as a loading control. **B.** Western blot result of indicated protein level before and after miR-9 expression in Huh-7 and SMMC7721. **C.** Western blot result of indicated protein level in SMMC7721 cells transfected with vector control, miR-9 only, or the combination of miR-9 and IGF2BP1.

The IGF2 mRNA binding protein family (IGF2BPs) is comprised of three members: IGF2BP1, IGF2BP2, and IGF2BP3, which are bona fide oncofetal proteins involved in various human cancers [19–21]. Interestingly, IGF2BP2 silencing has been proved to reduce ERK phosphorylation as its main mechanism of action [22]. Moreover, PI3K and MAPK are found to be essential for IGF2BP3 induced cell proliferation [23]. PI3K/AKT and ERK pathways are well known for their oncogenic properties [24, 25], and targeting them could well explain the tumor suppressive effect of miR-9 in HCC. Therefore, first we proceeded to see if IGF2BP1 could also induce ERK and AKT phosphorylation. As expected, western blot results showed that IGF2BP1 indeed induced phosphorylation of AKT (Ser473) and ERK (Thr202/Tyr204). GSK-3β is the substrate for both activated AKT and ERK. The observation of increased phospho-GSK-3β (Ser9), its inhibitory form, further confirmed that IGF2BP1 could activate PI3K/AKT and ERK pathways in SMMC7721 and Huh-7 cells (Figure 5A). Since we have already confirmed the capability of miR-9 to downregulate IGF2BP1, the potential suppressive effect of miR-9 on AKT and ERK phosphorylation in SMMC7721 and Huh-7 cells was also explored. Western blot results revealed that in both cell lines, restoration of miR-9 could block the phosphorylation of AKT (Ser473) and ERK (Thr202/Tyr204) as well as GSK-3β (Ser9) (Figure 5B). More importantly, exogenous IGF2BP1 expression could abrogate miR-9-mediated AKT, ERK and GSK-3β (Ser9) phosphorylation inhibition (Figure 5C), suggesting IGF2BP1 indeed plays a part in miR-9-induced ERK and AKT signaling pathway suppression. Taken together, our observations suggested that miR-9 plays a tumor suppressor role in HCC, partially via targeting IGF2BP1 to inhibit ERK and AKT oncogenic pathways.

## DISCUSSION

Hepatocellular carcinoma (HCC) is the most common primary liver malignancy. Although the deregulation of miR-9 in carcinogenesis has been discussed in several kinds of cancer, the function of miR-9 in cancer biology is still not well understood and the role of miR-9 in HCC has not been fully explored. In this study, by using a HCC patient cohort, we demonstrated that miR-9 was remarkably downregulated in HCC tissues, which was largely contributed by epigenetic silencing of mir-9-1. In addition, functional assays showed that miR-9 overexpression in HCC cell lines could inhibit their oncogenic properties *in vitro*. To mechanically understand the anti-tumor function of miR-9, we have identified a series of miR-9 target genes in HCC by comprehensively profiling its downstream candidate targets using multiple strategies. And some of the target genes are very likely to play key roles in HCC carcinogenesis evidenced by their enhanced expression in HCC tissues as well as their prognostic significances.

Two previous studies conducted in German population have suggested that mir-9-2 and mir-9-3 were frequently methylated in HCC [8, 26]. However, only hypermethylation of mir-9-1 was detected and was the main contributor to miR-9 downregulation in HCC tissues from Chinese population in our study, while no methylation of mir-9-2 and mir-9-3 was detected in HCC cell lines as well as 20 primary HCC tissues (data not shown). The cohort used in the first study consisted of 10 HCC cases and 15 fibrolamellar carcinoma cases, a rare variant of hepatocellular carcinoma. Among those cases, one was HBV positive and one was HCV positive [8]. In the second study, none of the 40 HCC cases was infected with HBV [26]. In contrast, over 80% patients in our cohort were chronic HBV infection-related. It is well known that HCC carcinogenesis is a complex process, which results in an obvious heterogeneity at both genetic and epigenetic levels [27]. Therefore, the discordance of miR-9 precursors' methylation statuses in HCC from different studies could be contributed by distinct etiologies, tumor origins, and backgrounds of the patient cohort.

MiR-9 has been reported to exert pro-metastasizing function in Sk-hep-1, which has a relatively high level of endogenous miR-9 [28]. However, in the current study, data derived from primary HCC specimens have suggested that the expression of miR-9 is downregulated in tumor tissues. The *in vitro* functional assay demonstrated an anti-tumor growth ability of ectopic miR-9 expression in HCC cell lines including SMMC7721, SNU449, and Huh-7 with very low endogenous miR-9 expression. Additionally, hypermethylation of mir-9-1 was found to be positively associated with larger primary tumor size in HCC patients. Taken together, all data here indicated a tumor suppressor role of miR-9 in HCC. These phenomena led us to hypothesize that in HCC cell lines as well as in the primary HCC tissues with low expression of miR-9, miR-9 exerts its tumor suppressive function by targeting a set of genes that are different from those in Sk-hep-1. On the other hand, unlike other HCC cell lines that are derived from hepatocellular carcinoma, Sk-hep-1 was derived from liver adenocarcinoma [29]; therefore the opposite function of miR-9 in Sk-hep-1 might also be caused by different cell origins.

We have identified seven miR-9 target genes (IL-6, AP3B1, TC10, ONECUT2, IGF2BP1, MYO1D, and ANXA2) in HCC. Among these seven genes, AP3B1, ONECUT2, and IL-6 were already confirmed to be the direct targets of miR-9 [15–17]; ANXA2 and IL-6 are well-established oncogenes in HCC [30–32]. These facts strongly supported the reliability of our selection strategy and indicated the importance of miR-9 in HCC carcinogenesis. The expression levels of ONECUT2, IGF2BP1, and ANXA2 were significantly upregulated in tumor tissues compared with non-tumor tissues, which suggested that they might play an oncogenic role in HCC development. However, our efforts to establish a significantly negative correlation between miR-9 expression level and the level of ONECUT2, IGF2BP1, and ANXA2 in HCC tissues were unsuccessful (data not shown). We know that the occurrence and development of HCC is a complicated process, during which a lot of aberrant events are involved, such as gene copy number variation, methylation and histone acetylation. Besides, microRNAs regulate their downstream targets in a rather delicate way, and each target usually has more than one upstream regulatory factor, so it's understandable that we couldn't establish such correlation between miR-9 and its targets in clinical samples. ONECUT2 is a member of the ONECUT transcription factor family that was shown to promote epithelial-mesenchymal transition (EMT), one of the well-defined processes during tumor invasion and distant metastasis [33]. Targeting ONECUT2 therefore inhibiting EMT could well explain miR-9 mediated migratory inhibition, but the detailed molecular mechanism still needs further study. Additionally, ONECUT2, IGF2BP1, and IL-6 upregulation predicted poor prognosis in the current study, which indicated that they might be potential prognostic biomarkers for applications in clinical practice.

Previous studies have reported that the IGF2 mRNA binding proteins (IGF2BPs) are powerful post-transcriptional oncogenes enhancing mesenchymal cell properties [34]. In HCC, IGF2BP1 could promote tumor cells proliferation by enhancing the expression of c-Myc, Ki-67 or PTEN/HSP27 [19, 20]. In this study, we provide first evidence that as an important pro-tumorigenic factor in HCC, IGF2BP1 was directly inhibited by miR-9 at its mRNA level. Furthermore, we speculated a novel mechanism in which re-expression of miR-9 could inhibit AKT and ERK phosphorylation by targeting IGF2BP1. More interestingly, the expression of IGF2BP1 was positively correlated with the level of AFP, the most widely recognized tumor marker for HCC. Whether IGF2BP1 can induce AFP expression in HCC and its underlying mechanism need to be further pursued in future study.

In the current study, we have proved that IL-6 is a direct target of miR-9 in HCC by demonstrating miR-9 could inhibit IL-6 at the translational level. IL-6 is a widely recognized risk factor in HCC, and several previous studies with a larger cohort size have demonstrated its diagnosis and prognostic predicting value in HCC [35, 36]. Consistently, our results here suggested that upregulation of IL-6 in HCC was positively correlated with later BCLC stage, and confirmed its value in predicting HCC patient post-surgery prognosis.

The current limitations of this study included the relatively small sample size in the prognosis analysis, lack of a patient cohort with chronic liver diseases as control, and short for cross-validation cohort. In China, over 80% of HCC patients are infected with HBV (mainly genotype B/C), which is different from the etiology of those in Europe and America. Therefore, further study should be conducted in larger HCC patient cohorts with different etiology, demography and geography.

Collectively, our preclinical results indicated that hypermethylation-mediated downregulation of miR-9 contributes to the proliferation and migration of HCC cells. The newly identified miR-9/IGF2BP1/AKT&ERK axis represents one of the anti-tumor mechanisms of miR-9 in HCC. The novel target genes of miR-9 have provided new insights into the pathogenesis of HCC, and they might serve as potential predictive and prognostic markers for HCC patients. Certainly, there are additional target genes contributing to the anti-tumor function of miR-9 in HCC; therefore, further effort should be made in the future to expand the miR-9 downstream functional network.

## MATERIALS AND METHODS

### Tissue samples

Eighty-seven pairs of primary human HCC tumor and the corresponding adjacent non-tumor tissue samples were obtained from patients undergoing surgical resection in Affiliated Oncology Hospital of Zhengzhou University from 2010 to 2014 (Supplementary Table S4), 16 normal liver tissues were from healthy liver donors. None of the patients in this study has received any kind of antiviral treatment before the liver resection surgery. All tissue samples were histologically confirmed and stored in liquid nitrogen. An informed consent was obtained from each patient and the study was approved by the Ethics Committee of Peking University Health Science Center.

### Cell culture

Human HCC cell lines SMMC7721, SNU182, Huh-7, and SNU449 cells, as well as human liver adenocarcinoma cell line Sk-hep-1, were grown in Dulbecco's modified Eagle's medium (DMEM) or RPMI-1640 supplemented with 10% fetal bovine serum (FBS, GIBCO, Carlsbad, CA, USA). All cell lines were maintained in a humidified incubator containing 5% CO_2_ at 37°C. Among all the cell lines we used, HBV DNA integration was only detected in SNU182 and SNU449, but their HBsAg levels were below detectable levels in cultured cell lysate as well as in the supernatant fluids [37].

### Methylation specific restrictive enzyme based quantitative PCR (MSRE-qPCR)

The quantificational methylation analysis was performed as previously described [13]. The primers used for MSRE-qPCR were listed in Supplementary Table S5. Methylation intensity (MI) was defined as the percentage of methylated target DNA sequences among all target DNA sequences. MI ≥ 10% was considered the criteria of hypermethylation [13].

### Real-time quantitative RT-PCR

Real-time qPCR was performed using the Roche LightCycler 480 sequence detection system (Roche, Mannheim, Germany). The expression of mature miR-9 was detected using the Taqman MicroRNA Assays (Applied Biosystems, Foster City, CA, USA) according to the manufacturer's protocol. Expression levels were normalized against the endogenous shRNA U6 control. For miR-9 target genes' mRNA expression, CTBP was used as an internal control. The relative expression level was calculated by the 2^−ΔCT^. The sequences of the RT-PCR primers were shown in Supplementary Table S6.

### Demethylation treatment of 5-aza-2′-deoxycytidine

SMMC7721 and SNU182 cells were seeded in 6-well plates at the concentration of 2.5 × 10^5^ cells per well. After 24 hours, cells were treated with DMSO control or 2 μmol/L 5-aza-2′-deoxycytidine, culture medium were changed with fresh medium containing drugs every 24 hours for a total of 3 days.

### Plasmid construction

To construct the miR-9 expression vector, human mir-9-1 gene and its 5′ and 3′ flanking region (120bp and 150bp, respectively) was amplified and cloned into pRNA-U6.1/Neo-siFluc to create the U6 driven mir-9-1, namely U6-mir-9-1. Stably miR-9 expressing HCC cell lines were established by transfection with the plasmid and then selection with G418. To construct pGL3-Luc-3′-UTR plasmids, 3′-UTR segments of predicted target genes containing the putative miR-9 binding site were amplified and cloned into a pGL3 vector downstream of *firefly* luciferase gene. Primers used for pGL3-Luc constructs were listed in Supplementary Table S7.

### Cell proliferation assay

Cells were seeded in 96-well plates at 2 × 10^3^ cells per well, and incubated at 37°C overnight. Then every day for a total of 5 days, 10 uL of CCK-8 (Dojindo Laboratories, Rockville, MA, USA) was added to each well, and the absorbance was measured at 450 nm one hour later using the microplate reader. The experiment was performed in sextuplicate. Background reading of medium was used to normalize the result.

### Wound healing assay

Stably transfected cells were cultured in 6-well plates until confluent. The cell layers were carefully wounded using a sterile 10 μL tip, washed twice and cultured in medium without FBS for 48 hours. Images of the wound monolayers were acquired with an optical microscope.

### Transwell migration assay

Cells were starved for 24 hours, and then 5 × 10^4^ cells were resuspended in 150 uL serum free media and then transferred to hanging cell culture inserts (Millipore Corporation, Billerica, MA). The inserts were then placed in each well of a 24-well plate containing 850 uL media with serum. After 48 hours, the inserts were washed with PBS and stained with crystal violet stain solution for 30 minutes. After rinsing with PBS and allowing the inserts to dry, an optical microscope was used to visualize the stained cells in random fields within each insert.

### Online prediction of target genes

Four bioinformatic softwares were used to predict potential target genes of miR-9 and are as following: TargetScan (http://www.targetscan.org/vert_60/), PicTar (http://pictar.mdc-berlin.de/), miRanda (http://www.microrna.org/) and miBridge.

### Luciferase reporter assay

Cells were seeded in a 12-well plate at 1 × 10^5^ cells per well and co-transfected with either U6-mir-9-1 or control plasmid along with pGL3-3′-UTR plasmids. pRL-TK was also transfected simultaneously as an endogenous control. Luciferase activity in each well was quantified 24 hours after transfection using Dual-Luciferase Reporter Assay System (Promega, Madison, WI, USA) according to the manufacturer's protocol. The relative luciferase activity was determined by dividing the *firefly* luciferase activity by the pRL-TK luciferase activity.

### ELISA assay

For quantitative detection of human interleukin-6 (IL-6) concentration in cell culture supernatants, cells were seeded and cultured in 6-well plates. After 48 hours, 200 uL culture supernatants were used for detection. Human IL-6 Quantikine ELISA Kit (R&D Systems, Minneapolis, Minnesota, USA) was used according to the manufacturer's introductions.

### Western blotting assay

Western blot assay was conducted as described before [38]. Anti-Erk1/2, anti-*p*-Erk1/2, anti-Akt, anti-*p*-Akt (Ser473), anti-GSK3β, and anti-*p*-GSK3β (Ser9) antibodies were obtained from Cell Signaling Technology (Cell Signaling Technology, MA, USA); anti-ONECUT2 antibodies were purchased from Santa Cruz (Santa Cruz, CA, USA); anti-GAPDH was bought from EASYBIO company (EASYBIO, Beijing, China); anti-mouse and anti-rabbit secondary antibodies conjugated with Cy5.5 were obtained from Amersham Pharmacia Biotech (Buckinghamshire, UK). The membranes were visualized using Odyssey Imager (LI-COR Biosciences, Lincoln, Neb, USA).

### Statistical analysis

A two-tailed Student's *t*-test was used to determine the statistical significances between two groups. Chi-square test or Fisher's exact test was used to determine the associations between gene expression status and patient clinicopathological features. Log-rank test was used for prognostic significance analysis. All statistical tests were two-sided. A *P* value of less than 0.05 was considered statistically significant. All statistical analysis was performed using the Statistical Analysis System (SAS 9.1).

## SUPPLEMENTARY FIGURE AND TABLES




