# Supplementary material for: Comprehensive profiling of novel microRNA-9 targets and a tumor suppressor role of microRNA-9 via targeting IGF2BP1 in hepatocellular carcinoma
Source: Oncotarget. 2015 Oct 19;6(39):42040–52. doi: 10.18632/oncotarget.5969 (PMC4747208; doi:10.18632/oncotarget.5969)
Supplement: Supplementary file 1 [file oncotarget-06-42040-s001.pdf]

SUPPLEMENTARY FIGURE AND TABLES

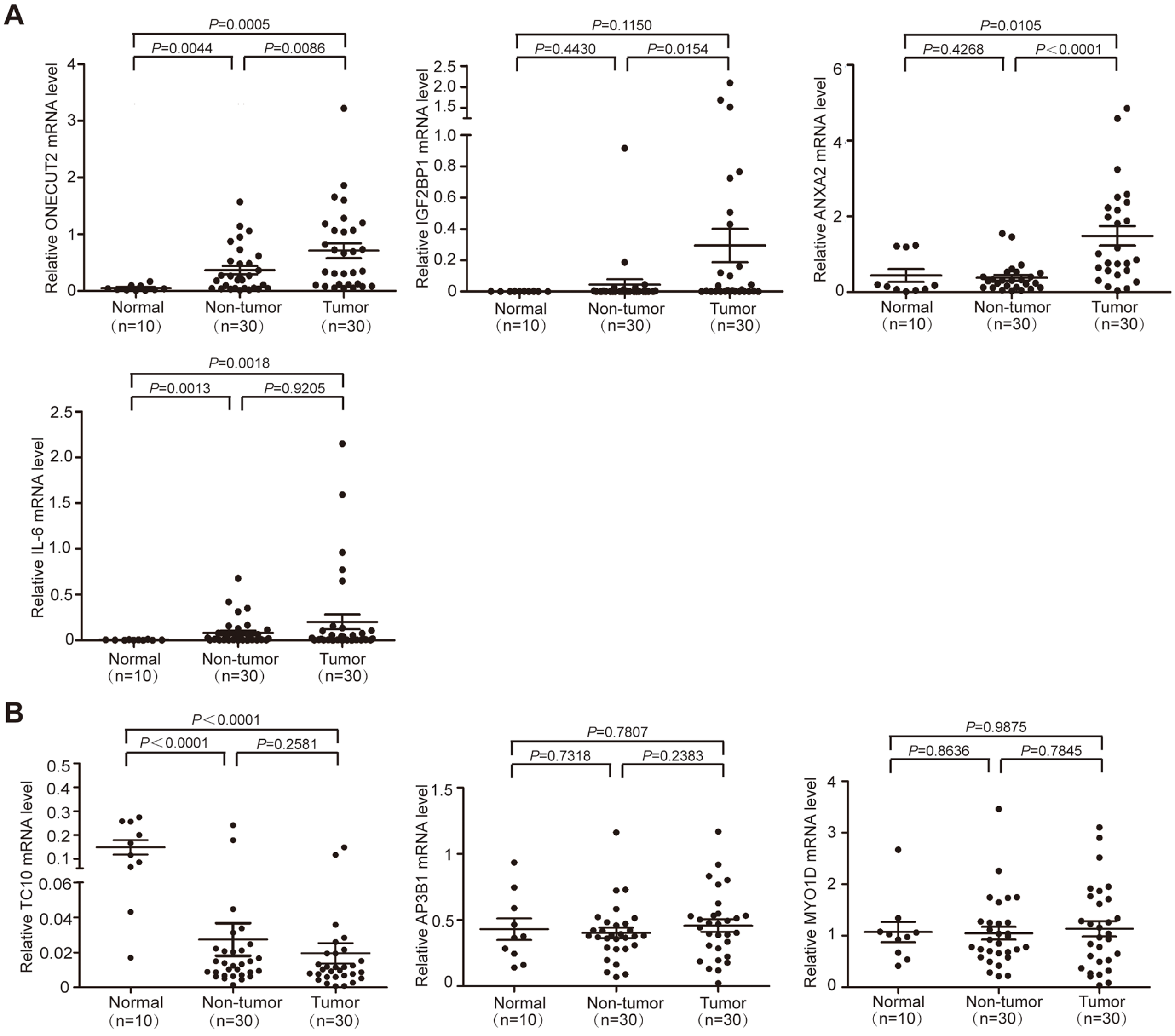

Supplementary Figure S1: A. Relative mRNA level of ONECUT2, IGF2BP1, ANXA2 and IL-6 genes in 30 paired HCC tumor tissues and adjacent non-tumor tissues, as well as 10 normal liver tissues. Data were represented as mean ± SEM. **B.** Relative mRNA level of TC10, AP3B1 and MYO1D genes in 30 paired HCC tumor tissues and adjacent non-tumor tissues, as well as 10 normal liver tissues. Data were represented as mean ± SEM.

Supplementary Table S1: miR-9 target gene list predicted by bioinformatic softwares.

Supplementary Table S2: Gene expression profiling in 6 pairs of primary HCC tissues.

Supplementary Table S3: The correlation between gene expression status and patients clinicopathologic features.

Supplementary Table S4: Clinicopathological features of HCC patients cohort

Clinicopathological Features		Cases *n* (%)
		n = 85*
Gender		
	Male	68 (80.00)
	Female	17 (20.00)
Age		
	Median (Range)	53 (21–76)
Cirrhosis		
	Yes	76 (89.41)
	No	7 (8.23)
	N/A	2 (2.35)
Portal Vein Invasion		
	Present	26 (67.06)
	Absent	57 (74.14)
	N/A	2 (2.35)
Tumor Size		
	≥5 cm	60 (70.59)
	<5 cm	17 (20.00)
	N/A	8 (9.41)
Tumor Encapsulation		
	Complete	68 (80.00)
	Uncomplete	9 (10.59)
	N/A	8 (9.41)
Intrahepatic Metastasis		
	Absent	34 (40.00)
	Present	49 (57.65)
	N/A	2 (2.35)
AFP		
	<400	40 (47.06)

(*Continued*)

**Clinicopathological Features**		**Cases *n* (%)**
	≥400	42 (49.41)
	N/A	3 (3.53)
Virus Infection Background		
	HBV	72 (84.7)
	HCV	2 (2.4)
	Others	11 (12.9)
HBsAg		
	Positive	72 (84.71)
	Negative	12 (14.12)
	N/A	1 (1.17)
HBeAg		
	Positive	2 (2.35)
	Negative	81 (95.30)
	N/A	2 (2.35)
BCLC		
	A	33 (38.82)
	B+C	49 (57.65)
	N/A	3 (3.53)
Survival		
	1 year	58 (68.33)
	2 year	47 (55.29)
	3 year	40 (46.67)
	4 year	28 (33.33)
	5 year	23 (26.67)
	N/A	22 (25.88)

Note: All patients were from the local ethnic Chinese Han population.
*The clinicopathological parameters of two patients are not avalible.

Supplementary Table S5: Primers sequences for the methylation detection and the size of the PCR products

Gene name	Forward (5′-3′)	Reverse (5′-3′)	Size (bp)
mir-9-1	CCGGCACCGCGGCTCC CCATTTCCAT	CTCCACTGCCCTTCTCTG GAAGATCGA	108
mir-9-2	TGTGTGCTGCGGCCGTGCTGTGA	TGCAGCCTGAGGGCCGCCACGATA	136
mir-9-3	CCGTGAACGCCGAGGCCATT	CGCGGAGCGCTTAGAGAGCC	222

Note: the annealing temperature of the primers are 74°C.

Supplementary Table S6: Primer sequences for the detection of target genes expression

Gene name	Forward (5′-3′)	Reverse (5′-3′)	Size (bp)
IL-6	TTCGGTACATCCTCGACGGCATC	CAGCTCTGGCTTGTTCCTCACTAC	258
AP3B1	GATGTTGAAGAGTGGGGGCA	TGTGCCAATACAGCTGAGCA	274
TC10	ACTGACCTTGATGGGTTTGG	TGGCCTTATTTGAGGCAGTG	331
ONECUT2	AGGCTGCCTACACCGCCTATC	GCCGTCCAGGATCGAGGCCAT	344
IGF2BP1	GCGATGAAGGCCATCGAAAC	AGCTTCATGATGGCTTGCCT	269
MYO1D	GAGCTGTATGAGAGACCGCC	CTGCTCTCTGACTGGGGTTG	181
ANXA2	TCCCGCTTGGTTGAACACAT	CATTGCTGCGGTTGGTCAAA	240
CTBP	TTCACCGTCAAGCAGATGAGAC	CTGGCTAAAGCTGAAGGGTTCC	156

Note: the annealing temperature of the primers are 60°C.

Supplementary Table S7: Primer sequences for luciferase reporter plasmid construction

Gene name	Forward (5′-3′)	Reverse (5′-3′)
**IL6-3'UTR**	GGGCCCGAGGGCTCTTCGGCAAAT	CTCGAGCTGGCTCTGAAACAAAGGATA
**AP3B1-3'UTR**	GGGCCCCCTGTCCTGTCTCAGGGGTA	CTCGAGGAGAAAACGCCACATGGATT
**TC10-3'UTR**	GGGCCCACTGACCTTGATGGGTTTGG	CTCGAGTGGCCTTATTTGAGGCAGTG
**ONECUT2-3'UTR**	GGGCCCGGGTTTTTCCTCATCCATCC	CTCGAGTCACATGAAAACGAGGCTGT
**IGF2BP1-3'UTR**	GGGCCCCCCTCCTCCCAGTCCTTATC	CTCGAGTCGAAAGCTGGCTATTGCTT
**MYO1D-3'UTR**	GGGCCCCGCCCCTGTAAGATTTCCTT	CTCGAGTGTCCAGGTGCTCTGACTTG
**ANXA2-3'UTR**	GGGCCCCTGTGAGGGTGACGTTAGCA	CTCGAGGAAAGCAGGGCCACAAAGTA
**MTHFD1L-3'UTR**	GGGCCCCGATGCAGACTCCTGAAACA	CTCGAGGCCGAACACCATACTCCACT
**HSPC159-3'UTR**	GGGCCCGAGGCACTGAAGTGACCATGT	CTCGAGCACGGAACAGGCTGAATATG
**KIF23-3'UTR**	GGGCCCCACGGACCTCAGCTACATCA	CTCGAGTCATCTAAGACAAACACTTGTGTGAA
**GLS-3'UTR**	GGGCCCGCCATGGAGACAGGTAGCAT	CTCGAGGAGCGCTCAGGATACAGAAAA
**HLTF-3'UTR**	GGGCCCTCAGACCTTTTATACTTTTGACCA	CTCGAGGGAAAAATAAAAACCTGTGCTAACC
**SGOL1-3'UTR**	CAGGGTTTCTCCATGTTGGT	GGGAAAAATAGAGATGCCAGAA
**LMNA-3'UTR**	GGGCCCTTCTCCTCCCTTCCTTTTCC	CTCGAGGGCTCAAGCTGTCCTCTCTC
**EHF-3'UTR**	GGGCCCTTTATTTCTGCCTGGCTTGG	CTCGAGACAGTCAAATGCTGGGAAGG
**PIK3R3-3'UTR**	GGGCCCCCCACGAAGAAGGCTGTTTA	CTCGAGAAACAGGCAGGAAGGACAGA
**FAM13C-3'UTR**	GGGCCCGGACACTTGTTAGGGAA	CTCGAGGTAAACCAAAAGATTAGC
**IGF2BP3-3'UTR**	GGGCCCCAGGTAAAGCAGCACCAACA	CTCGAGAGTTGCCTGGTCCTCAGAAA
**RAB11FIP4-3'UTR**	GGGCCCGCTACCTACCTCCCCGTTTC	CTCGAGCTGCGTGGAGAATGAAGACA

Note: the annealing temperature of the primers are 57–65°C.
